# Fabrication of magnetic water-soluble hyperbranched polyol functionalized graphene oxide for high-efficiency water remediation

**DOI:** 10.1038/srep28924

**Published:** 2016-06-29

**Authors:** Lihua Hu, Yan Li, Xuefei Zhang, Yaoguang Wang, Limei Cui, Qin Wei, Hongmin Ma, Liangguo Yan, Bin Du

**Affiliations:** 1 Key Laboratory of Chemical Sensing & Analysis in Universities of Shandong, School of Chemistry and Chemical Engineering, University of Jinan, Jinan 250022, PR China; 2 School of Resources and Environment, University of Jinan, Jinan 250022, PR China

## Abstract

Magnetic water-soluble hyperbranched polyol functionalized graphene oxide nanocomposite (MWHPO-GO) was successfully prepared and applied to water remediation in this paper. MWHPO-GO was characterized by Fourier transform infrared spectroscopy (FTIR), X-ray diffraction (XRD), thermogravimetric analysis (TGA), magnetization curve, zeta potential, scanning electron microscope (SEM) and transmission electron microscope (TEM) analyses. MWHPO-GO exhibited excellent adsorption performance for the removal of synthetic dyes (methylene blue (MB) and methyl violet (MV)) and heavy metal (Pb(II)). Moreover, MWHPO-GO could be simply recovered from water with magnetic separation. The pseudo-second order equation and the Langmuir model exhibited good correlation with the adsorption kinetic and isotherm data, respectively, for these three pollutants. The thermodynamic results (Δ*G* < 0, Δ*H* < 0, Δ*S* < 0) implied that the adsorption process of MB, MV and Pb(II) was feasible, exothermic and spontaneous in nature. A possible adsorption mechanism has been proposed where π-π stacking interactions, H-bonding interaction and electrostatic attraction dominated the adsorption of MB/MV and chelation and electrostatic attraction dominated the adsorption of Pb(II). In addition, the excellent reproducibility endowed MWHPO-GO with the potential for application in water remediation.

Water pollution with toxic synthetic dyes and heavy metal ions has been a serious environmental hazard for years along with developing of modern industry and agriculture. Therefore, the decontamination of wastewater containing these pollutants has caused a great of concern^1^. The plastics, leather, papermaking and textile industries are some common sources of dye effluents. Dyes with a complex aromatic structure are more stable and difficult to biodegrade. Methylene blue (MB) and methyl violet (MV), which are the most commonly used substance for dying silk, wood or cotton, may cause neurological injury, nausea and vomiting^2^. Mining and metallurgical industries are the main sources of heavy metals effluents. In context of the toxic metals, lead is considered to be the typical one, which can cause sickness, neurological disorders, kidney disease and even death in humans after frequent contact^3^. Due to the strong toxicity, nonbiodegradability and accumulation in plants, animals and human beings, synthetic dyes and heavy metals should be cleaned from wastewater prior to being released into the environment. Numerous chemical, physical and biological treatment methods have been developed to treat the wastewater in recent years. Among these methods, the adsorption technique is especially attractive due to its ease of operation, high efficiency and availability for many pollutants^4^. However, the traditional adsorbents, such as zeolite^5^, activated carbon^6^, and polymeric materials^7^, are typically limited by low adsorption capacities or recycling problems. Nanostructured materials with high surface area and special properties have been widely explored as high-efficiency sorbents. However, the difficulty of solid–liquid separation limits their practical application. Therefore, the development of new adsorbents with better adsorption performance and separation property is highly desirable.

Magnetic nanoparticles (MNPs) can be facilely separated from sample solution by applying an external magnetic field, which affords a rapid and economic approach to remove the toxic compounds from large-volume samples. Therefore, numerous studies have been devoted to the MNP adsorption. Among MNPs, Fe_3_O_4_ nanocomposites have been widely investigated to study the removal of dyes and toxic heavy metals from wastewater^8^. However, the adsorption capacities of these magnetic adsorbents are relatively low because of the poor water dispersity and low density of surface functional groups. It is known that functional groups such as hydroxyl, amine, thiol and carbonyl have strong interactions with various synthetic dyes and heavy metals^9^,^10^,^11^. Therefore, water-soluble polymers containing many desired functional groups are typically chosen to modify Fe_3_O_4_ nanoparticles to improve dispersion properties in aqueous solutions as well as the adsorption capacity. For example, Zhou *et al*.^12^ fabricated ethylenediamine-modified magnetic chitosan nanoparticles, which were used for the removal of organic dyes with a very high capacity. Ge and co-workers^13^ prepared novel Fe_3_O_4_@APS@AA-co-CA MNPs nanoparticles and found these particles could efficiently remove the heavy metal ions such as Cd^2+^, Zn^2+^, Pb^2+^ and Cu^2+^ from aqueous solution with high maximum adsorption capacity.

As an emerging carbon nanomaterial, graphene has been used as an excellent adsorbent to remove a range of benzenoid contaminants and heavy metals because of its very large specific surface areas and other unique properties^14^. However, due to the scarcity of surface functional groups and aggregation via van der Waals interactions, the adsorption capacities of graphene nanoadsorbents is limited^15^. To solve this problem, graphene is often functionalized with inorganic nanoparticles, surfactants, hydrophilic groups, or polymers^16^,^17^,^18^.

Due to the nearly spherical structure and numerous surface functional groups, hyperbranched polymers (HPs) are more suitable for the modification of nanomaterials compared with traditional linear polymers. Moreover, HP has weak or even non-existent molecular chain entanglements, so most of the functional groups can closely contact with adsorbates^19^. Recently, researchers reported that HP modified substrates, such as magnetic particles^20^, silica-gels^21^, and collagen fibers^22^, realized effective removal of synthetic dyes and heavy metals. But as far as we know, no studies have employed magnetic hyperbranched polyol modified graphene oxide (GO) as an adsorbent.

In this study, Fe_3_O_4_ magnetic particles, water-soluble multifunctional hyperbranched polyol (WHPO) were combined with high surface area GO to prepare a novel nanosorbent (MWHPO-GO) for the high efficiency removal of both synthetic dyes and heavy metals. The obtained MWHPO exhibited good water solubility and strong affinity toward the pollutants because of the abundant hydroxyl, ether and amine groups. Therefore, anchoring of MWHPO onto the GO surface is beneficial for improving the adsorption capacities of pollutants. The synthesized MWHPO-GO was characterized by FTIR, TGA, XRD, zeta potential and SEM analyses. The batch adsorption tests of MB, MV and Pb(II) by MWHPO-GO were carried out to study the adsorption kinetics, isotherms and thermodynamics and a possible adsorption mechanism has been proposed. In addition, to further evaluating the practical applications, the effect of coexisting ions and the regeneration performance of MWHPO-GO were also investigated.

## Materials and Methods

### Chemicals and materials

All reagents used in the experiment were of analytical reagent grade. Poly(ethylene oxide) diglycidyl ether (PEO-DE, Sigma Aldrich, USA) and N-ethylethylene diamine (EED, Alfa Aesar, London) were used as received. 3-Isocyanatomethyl-3,5,5-trimethylcyclohexyl isocyanate (IPDI), dibutyltin dilaurate (DBTDL), 1-ethyl-3-(3-dimethylaminopropyl) carbodiimide hydrochloride (EDC), N-hydroxyl succinimide (NHS) and other chemicals were obtained from Sinopharm Chemical Reagent Beijing Co., Ltd, China. In addition, methylene blue (MB) and methylene violet (MV), which were employed as the dye source, and lead nitrate, which was employed as the heavy metal source, were dissolved in ultrapure water prior to use. 0.1 mol L^−1^ HCl and 0.1 mol L^−1^ NaOH were used for pH adjustment.

### Synthesis of water-soluble hyperbranched polyol (WHPO)

WHPO was synthesized through the nucleophilic ring-opening reaction of diepoxy and diamine monomer^23^. The reaction was conducted in a three-neck flask equipped with a nitrogen inlet tube and a reflux condenser. PEO-DE (0.04 mol), EED (0.04 mol) and ethanol (120 mL) were mixed and stirred at room temperature for 48 h and then refluxed for an additional 24 h. The concentrated solution was then precipitated in n-hexane to produce a viscous liquid followed by drying in a vacuum oven at 50 °C for 24 h. The yield of the light yellow liquid WHPO was approximately 90%.

### Synthesis of magnetic WHPO (MWHPO)

Fe_3_O_4_ was prepared by the traditional hydrothermal method^24^. Magnetic WHPO was synthesized according to the following steps: First, Fe_3_O_4_ (200 mg) and DBTDL (50 mg) was fully dispersed in 200 mL of toulene. Then, IPDI (3 g) was added drop by drop, and the mixture was vigorously stirred at 45 °C for 12 h. Then the solid was separated from the solution through magnetic separation. After washing four times by toluene, the solid (named Fe_3_O_4_-IPDI) and DBTDL (50 mg) were dispersed in 250 mL of toluene. Next, WHPO (1 g) was added slowly into the mixture and the solution was stirred at 60 °C for 12 h. Finally, the resulting product was separated and repeatedly washed with toluene three times to remove the free WHPO polymers that were not anchored to the Fe_3_O_4_ magnetic particles. The product (MWHPO) was dried in vacuum at 50 °C for 24 h.

### Synthesis of MWHPO-modified grapheme oxide (MWHPO-GO)

Graphene oxide (GO) was prepared from purified natural graphite using the modified Hummers method^25^. GO (100 mg), EDC (120 mg) and NHS (250 mg) were dispersed in 200 mL of phosphate buffer saline (pH = 7.4). After the obtained mixture was stirred at room temperature for 2 h, MWHPO (200 mg, the weight ratios of MWHPO to GO was 2:1) was added and the reaction was continued at room temperature for 6 h. The magnetic solid was separated and washed with water three times to get rid of the unmodified GO. The final product, named MWHPO-GO, was dried in vacuum at 50 °C for 24 h.

In addition, other weight ratios of MWHPO to GO were applied to prepare a series of adsorbents with different component according to the similar synthetic method. The designed other weight ratios of MWHPO to GO were 1:2, 3:1, 5:1, and the corresponding products were named MWHPO-GO-0.5, MWHPO-GO-3, MWHPO-GO-5, respectively.

### General characterization

The structure and performance of the synthesized materials were characterized by several techniques. The FTIR spectra measurements were mounted by using a Perkin-Elmer Spectrum One FTIR spectrometer (Perkin-Elmer, United States) in KBr pellet at room temperature in a spectral range of 4000–400 cm^−1^. ^1^H nuclear magnetic resonance spectroscopy (^1^H NMR) was recorded in a DMX-300 MHz instrument (Bruker, Germany) using CDCl_3_ as the solvent. The XRD patterns were acquired on a RigakuD/MAX 2200 X-ray diffractometer (Tokyo, Japan). TGA was performed on a Diamond High Temperature Type TG/DTA thermoanalyzer (Perkin-Elmer, United States) under a N_2_ atmosphere from room temperature to 800 °C with heating rate of 10 °C min^−1^. The magnetization curves of samples were measured through MPMS3 system on superconducting quantum interference device (Quantum Design, USA) at 300 K. For zeta potential analysis, 10 mg of Fe_3_O_4_, MWHPO or MWHPO-GO powder was dispersed in 25 mL of ultrapure water with various pH values, respectively. The obtained solution samples were measured with a JS94H (Shanghai, China). SEM and energy-dispersive X-ray spectroscopy (EDX) were performed using a FEI QUANTA FEG250 coupled with INCA Energy X-MAX-50. Transmission electron microscopy (TEM) images were obtained from a JEOL JEM-100CX II.

### Adsorption experiments

To assess the adsorption performance, batch adsorption experiments were performed by taking MB, MV and Pb(NO_3_)_2_ as probes. Water samples were prepared by dissolving known amounts of heavy metal or organic dyes in ultrapure water. The prepared adsorbent (MWHPO-GO) was placed in a beaker containing different volume of MB (25 mL), MV (25 mL) or Pb(II) (10 mL) aqueous solution and then shaken on a temperature-controlled shaker. Optimization of adsorbent components were carried out by treating 25 mL, 20 mg L^−1^ of MB or Pb(II) solution with 10 mg of adsorbent (contact time was 3 h, temperature was 298 K). The dosage effect was tested in the 1–8 mg range for MB, 1–14 mg range for MV and 2–12 mg range for Pb(II) (*C*_0_ = 30 mg L^−1^, contact time was 3 h, temperature was 298 K). The effect of pH was studied in the range of 2–9 for MB, 2–9 for MV and 2–7 for Pb(II). The effect of the contact time was determined from 10–180 min for MB, 10–180 min for MV and 10–150 min for Pb(II). (pH = 6, temperature was 298 K, 6 mg dosage was used for MB, 10 mg dosage was separately used for MV and Pb(II)). The adsorption equilibrium isotherms were determined with an initial concentration range of 5–300 mg L^−1^ for MB, 10–200 mg L^−1^ for MV and 30–120 mg L^−1^ for Pb(II). The adsorption thermodynamics were studied at temperatures ranging from 298 to 318 K with varying initial concentrations.

At the end of the adsorption, the adsorbent was isolated by an external magnet, and the supernatant was collected to determine the residual concentrations of pollutants. The removal efficiency and the amount of pollutants adsorbed onto MWHPO-GO were calculated using the following equations:


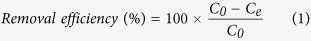



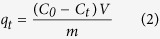


where *C*_*0*_ and *C*_*e*_ (mg L^−1^) are the initial and equilibrium concentrations of the pollutant, respectively. *C*_*t*_ (mg L^−1^) is the concentration of adsorbate in the aqueous solution at time *t* (min). *q*_*t*_ (mg g^−1^) is the amount of adsorbate adsorbed per unit mass of the adsorbent at time *t*. *V* (L) is the volume of the adsorbed solution, and *m* (g) is the mass of the adsorbent.

### Regeneration of the adsorbent

In the desorption experiments, an ethanol (25 mL) and HCl solution (25 mL, 2 M) were used as the desorption agents to regenerate the adsorbents from the MWHPO-GO loaded with organic dyes and heavy metal samples, respectively. After shaking for 180 min at 298 K, the samples that separated from the solution by magnet were washed three times with ultrapure water and subjected to the next adsorption-desorption process to recycle. The adsorption-desorption cycle was successively conducted 5 times for each test.

### Replication of batch experiment

Each batch adsorption experiment was conducted twice and the data shown are the average values. The individual values were generally within 5%.

## Results and Discussion

### General characterization

**Figure 1:**
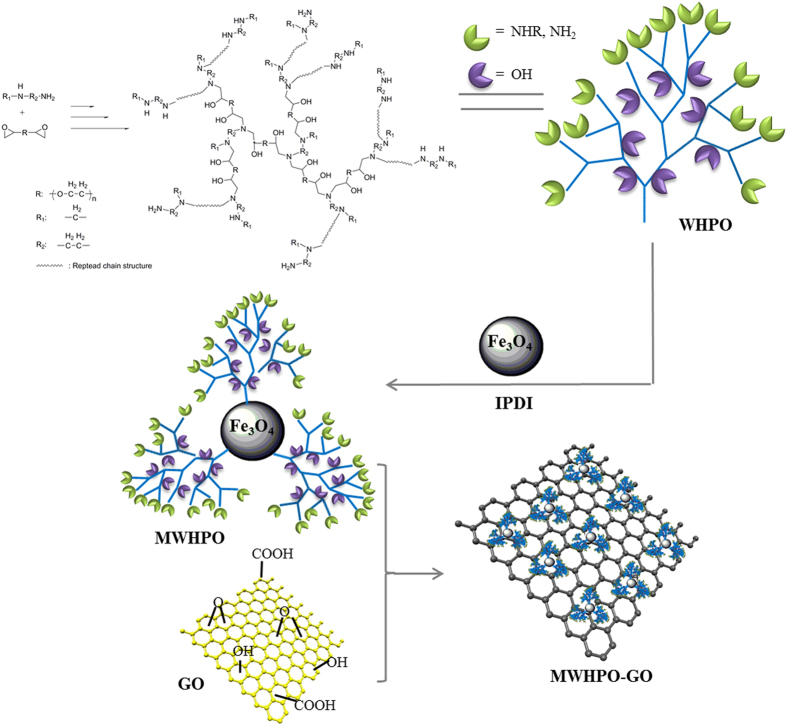
The preparation process of MWHPO-GO nanoadsorbent.

**Figure 2:**
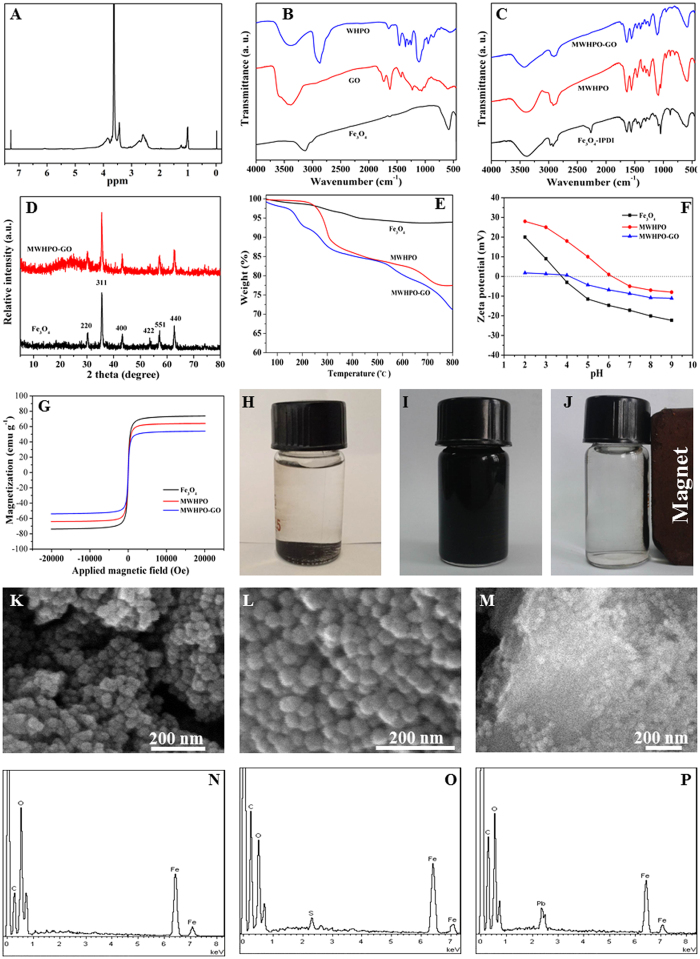
Characterization of as-prepared adsorbent: ^1^H NMR spectrum of WHPO in CDCl_3_ (**A**); FTIR spectra (**B,C**); XRD patterns of Fe_3_O_4_ and MWHPO-GO (**D**); TGA analysis (**E**), Zeta potential of Fe_3_O_4_, MWHPO and MWHPO-GO (**F**); Magnetization curves (**G**); photographs for the dispersion status of Fe_3_O_4_ (**H**) and MWHPO-GO (**I**) in water settled for 2 h and 5 days, respectively; Demonstration of magnetic separation of MWHPO-GO (**J**); SEM images of Fe_3_O_4_ (**K**), MWHPO (**L**) and MWHPO-GO (**M**); EDX analysis of MWHPO-GO before (**N**) and after loading with MB (MWHPO -GO-MB, (**O**) and Pb(II) (MWHPO-GO-Pb(II) (**P**).

Figure 1 illustrates the preparation process of MWHPO-GO and its subsequent adsorption of several pollutants. WHPO can be obtained through a one-pot reaction and its molecular structure was determined using the ^1^H NMR spectrum (Fig. 2A). The characteristic peaks at 3.3–4.1 ppm and 2.3–3.0 ppm corresponded to the signal from -CH_2_- and -CH- connected to oxygen and nitrogen atoms, respectively. In addition, the peaks of the methyl groups appeared at 0.9–1.3 ppm. These results indicated the successful synthesis of WHPO according to the literature^23^. In order to endow the composite adsorbent with well magnetic separation property, Fe_3_O_4_ magnetic particles were coupled with WHPO by using IPDI to generate MWHPO. Then MWHPO was grafted onto GO surfaces through the reaction between amine groups of MWHPO and carboxyl groups of GO, which could introduce hydroxyl and amine groups onto the GO surface and increase the adsorption efficiency of dyes and heavy metals. In addition, due to the good water solubility of WHPO grafted onto the GO surface, it was well dispersed in the aqueous solution, which was helpful for improving the adsorption properties of MWHPO-GO.

The covalent functionalization of graphene oxide with MWHPO was characterized by FTIR spectroscopy (Fig. 2B,C). In the Fe_3_O_4_ spectrum, the strong IR band at 581 cm^−1^ is characteristic of the Fe-O vibrations^26^, while the transmissions around 1625 and 3120 cm^−1^ matched well with that from the free -NH_2_ group on the Fe_3_O_4_ particles^27^. The characteristic peaks in the GO spectrum at 3391, 1738, 1633, 1434 and 1229 cm^−1^ were due to the OH, C = O in -COOH, aromatic C = C, carboxy C-O, and epoxy C-O stretches, respectively^28^. In the WHPO spectrum, the broad adsorption band located at 3404 cm^−1^ was due to the stretching vibration of O-H and N-H. The adsorption band located at 2875 cm^−1^ corresponded to the stretching of the -CH_2_- groups. The peak at 1652 cm^−1^ corresponded to N-H bending vibration of -NH_2_^29^. The characteristic peak of the aliphatic C-O ether bond appeared at 1100 cm^−1^.

IPDI was selected as the coupling agent to combine Fe_3_O_4_ and WHPO due to the existence of two –NCO groups with different reaction activities in IPDI molecule^30^. When Fe_3_O_4_ particles reacted with greatly excess IPDI by using DBTDL as a catalyst, the secondary cycloaliphatic isocyanate groups were more reactive to react with amine groups. So the primary isocyanatomethyl groups in IPDI molecules could be left for subsequent reactions. As shown in the Fe_3_O_4_-IPDI spectrum (Fig. 2C), new peaks located at 1639 cm^−1^ and 1559 cm^−1^ was assigned to the carbamido groups (-NH-CO-NH-), suggesting the successful reaction of the amine groups on Fe_3_O_4_ with the isocyanate groups of IPDI. In addition, a new peak located at 2267 cm^−1^ was from -NCO groups, and two new bands appeared at 2842 cm^−1^ and 2975 cm^−1^ due to -CH_2_- stretching vibrations of IPDI, which also indicated the effective modification of Fe_3_O_4_ by IPDI.

In the MWHPO spectrum (Fig. 2C), the peak located at 2267 cm^−1^ that was assigned to -NCO groups disappeared, and a new peak at 1095 cm^−1^ came from the ether groups of WHPO appeared, which implied that Fe_3_O_4_ and WHPO were combined successfully through IPDI to obtain magnetic WHPO (MWHPO). Compared with the GO spectrum, the intensity of the peak located at 1738 cm^−1^ assigned to the carboxyl groups in GO surfaces decreased in the MWHPO-GO spectrum (Fig. 2C), indicating the successful grafting of MWHPO onto the GO surface. In addition, the peak located at 1635 cm^−1^ was due to vibration of the aromatic rings and amine groups. It is important to note that the abundant hydroxyl and amine groups in WHPO not only can interact with organic dyes and heavy metals, but also endow WHPO with good water solubility, which is beneficial for improving the water dispersity of Fe_3_O_4_ and GO. So the designed MWHPO-GO nanosorbents are expected to remove dyes and heavy metals with high capacities.

The wide-angle XRD patterns of Fe_3_O_4_ and MWHPO-GO are shown in Fig. 2D. The diffraction peaks at 30.1 °, 35.5 °, 43.1 °, 53.4 °, 57.3 ° and 62.6 ° represented the (220), (311), (400), (422), (551) and (440) lattice planes of Fe_3_O_4_ according to the JCPDS card (JCPDS 89–4319)^31^. The sharp, strong peaks confirmed the Fe_3_O_4_ was well crystallized. These characteristic peaks of Fe_3_O_4_ also appeared at the pattern of MWHPO-GO, indicating that MWHPO-GO had retained the spinel structure of Fe_3_O_4_. In addition, a broad diffraction peak corresponding to amorphous WHPO appeared in the pattern of MWHPO-GO. The results from the XRD analysis also indicated that MWHPO was anchored onto the GO nanosheets.

The weight loss curves for Fe_3_O_4_, MWHPO and MWHPO-GO obtained from TGA are shown in Fig. 2E. The weight loss of Fe_3_O_4_ throughout the heating process was about 6.2%, which was due to the elimination of absorbed water and degradation of surface organic components. When WHPO was modified onto Fe_3_O_4_ particles surface, the obtained MWHPO exhibited a weight loss stage from 210 °C to 750 °C, and the residue at 800 °C was 77.8%, which was lower than that of Fe_3_O_4_ particles (93.8%) due to the loss of WHPO components. In the MWHPO-GO curve, the weight loss started below 120 °C due to the volatilization of adsorbed water aroused by GO. The residue at 800 °C was 71.2%, which was lower than that of MWHPO. This was because MWHPO-GO possessed more organic components came from WHPO and GO. These TGA results indicated that MWHPO has been successfully grafted onto the GO surfaces, and the magnetic nanocomposite adsorbent exhibited good thermal stability, which is beneficial for the application of the adsorbent.

The electronic charges on the surface of adsorbents in aqueous solutions can be analyzed by zeta potentials^32^. As shown in Fig. 2F, the point of zero charge (pHzpc) of MWHPO-GO was 4.1. This result indicated that when the pH was higher than 4.1, the surface of MWHPO-GO was negatively charged due to the deprotonation of the hydroxyl groups and carboxyl groups. Since electrostatic interactions usually dominated the adsorption process of cationic pollutants, such as heavy metal ions and cationic dyes, MWHPO-GO is expected to exhibit increased adsorption capacities in more alkaline conditions.

The hysteresis loops of Fe_3_O_4_, MWHPO and MWHPO-GO at 300 K were demonstrated in Fig. 2G. Due to the modification of WHPO and GO, the saturation magnetization values of MWHPO (64.1 emu g^−1^) and MWHPO-GO (54.1 emu g^−1^) were lower than that of pure Fe_3_O_4_ (73.8 emu g^−1^). However, the saturation magnetization value of MWHPO-GO was strong enough to achieve a facile magnetic separation as shown in Fig. 2J.

Due to the introduction of water-soluble HPO, MWHPO-GO was easily dispersed in water using ultrasonication. Figure 2H and I show the dispersion state of pure Fe_3_O_4_ and MWHPO-GO in water (2 mg mL^−1^) at room temperature after settling for different periods of time. MWHPO-GO can be uniformly dispersed in water to form homogenous and stable solutions, and then, the dispersions were allowed to settle for at least five days without obvious precipitants (Fig. 2I). However, the pure Fe_3_O_4_ cannot be well dispersed and precipitates formed in 2 h after ultrasonication (Fig. 2H). The well dispersed MWHPO-GO can closely contact with the contaminants, which would be beneficial for the high-efficient water treatment. In addition, MWHPO-GO can be rapidly collected within 20 seconds from an aqueous suspension by an external magnetic field (Fig. 2J). After that, the separated composites were easily redispersed with ultrasonication to a fairly stable suspension. This is essentially important for the convenient reuse of MWHPO-GO.

The SEM images showed the microstructure of Fe_3_O_4_ (Fig. 2K and Fig. S1A), MWHPO (Fig. 2L and Fig. S1B) and MWHPO-GO (Fig. 2M and Fig. S1C). The average diameter of the particles was estimated by Nano Measurer soft. Pure Fe_3_O_4_ particles showed a certain degree of aggregation with the average size of about 35 nm (Fig. 2K and Fig. S1A). After successful grafting of WHPO polymers, the resulted MWHPO particles showed a better dispersion state and bigger average size of about 43 nm (Fig. 2L and Fig. S1b). GO was reported as a single atomic sheet of sp^2^-hybridized carbon atoms, it has ultrahigh specific surface area and opened a new avenue for preparing composite materials^33^. SEM images of the as-prepared MWHPO-GO (Fig. 2M and Fig. S1C) displayed that MWHPO particles were successfully anchored onto the surface of GO. TEM images showed in Supplementary Fig. S1 online further characterized the morphology structure of the prepared materials. The abundant functional groups and high specific surface area made the composite adsorbent MWHPO-GO had a great potential for water treatment.

The elemental composition of the sorbent before and after adsorption was analyzed through EDX analysis. According to the results shown in Fig. 2N, C, O and Fe existed in the spectrum of MWHPO-GO prior to adsorption. After adsorption of MB and Pb(II) onto MWHPO-GO, new peaks corresponding to Pb and S appeared in the spectrum of MWHPO-GO-MB (Fig. 2O) and MWHPO-GO-Pb(II) (Fig. 2P), respectively. These results qualitatively indicate that MB and Pb(II) were successfully adsorbed onto MWHPO-GO. In addition, the weight percentage of elements corresponding to Fig. 2N–P has been listed in Supplementary Table S1 online. For MWHPO-MB, C element content increased obviously due to the adsorption of organic carbon compound, MB.

### Optimization of adsorbent components

Different component adsorbents usually show different adsorption performances. For the sake of comparison, MWHPO (number 1), MWHPO-GO-0.5 (number 2), MWHPO-GO (number 3), MWHPO-GO-3 (number 4) and MWHPO-GO-5 (number 5) were separately used to remove MB and Pb(II). As shown in Supplementary Fig. S2 online, all the adsorbents containing GO showed a higher adsorption capacity for MB and Pb(II) compared with that of pure MWHPO, which was due to that GO component endowed the nanocomposite adsorbent large surface area. In addition, the increase of GO contents of the nanocomposite adsorbents resulted increased adsorption capacities of MB and Pb(II) (MWHPO-GO-0.5, MWHPO-GO). However, when the weight ratios of MWHPO to GO were higher than 2:1, the generated nanocomposite adsorbents (MWHPO-GO-3, MWHPO-GO-5) gave decreased adsorption capacities of the pollutants. This was due to that too many GO sheets might cap MWHPO particles, which impeded the contact of adsorption sites with the pollutants.

### Effect of dosage on the adsorption behavior

**Figure 3:**
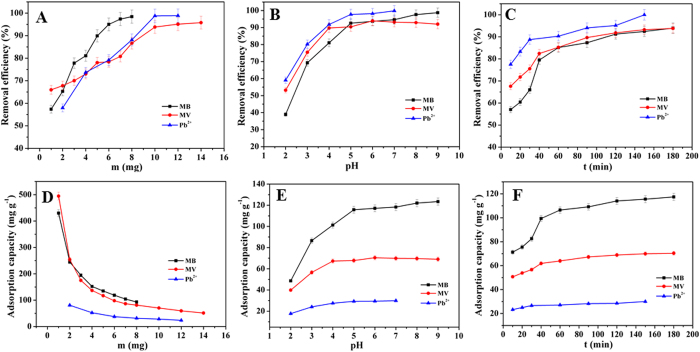
Effect of dosage (**A,D**), pH (**B,E**) and contact time (**C,F**) on adsorption behavior of MB, MV (*C*_0_ = 30 mg L^−1^, *V* = 25 mL, temperature was 298 K) and Pb(II) (*C*_0_ = 30 mg L^−1^, *V* = 10 mL, temperature was 298 K). Error bar = SD (n = 2).

The effect of the adsorbent dosage on the removal efficiency and adsorption capacity was investigated by adding various amounts of the MWHPO-GO nanocomposite to MB, MV and Pb(II) solutions followed by shaking at room temperature for 3 h (Fig. 3A,D). The removal efficiency of the three contaminants increased as the adsorbent dosage increased, which was due to more adsorption sites being available at higher adsorbent dosages. However, when the adsorption process reaches a saturated state, no more contaminants can be adsorbed onto the adsorbent even if the dosage of the adsorbent is increased. As indicated by the results, the removal efficiency reached an equilibrium at 95% for MB at 6 mg MWHPO-GO dosage, 93.8% and 98.8% for MV and Pb(II), respectively, corresponding to 10 mg MWHPO-GO dosage. Considering the removal efficiency and practicality, the optimal adsorbent dosage was maintained at 6 mg for MB, 10 mg for MV and Pb(II) in all subsequent experiments.

### Effect of pH on the adsorption behavior

Since aqueous solution pH has been reported as an important factor that affects the removal efficiency of dyes and heavy metal ions^34^, batch equilibrium experiments were carried out to confirm the effect of pH on solution adsorption by MWHPO-GO in a wide range of pH values. The uptake of MB, MV and Pb(II) onto MWHPO-GO as a function of the corresponding solution pH are shown in Fig. 3B,E. According to the obtained zeta potential results, as the pH values increased, the surface charge of MWHPO-GO became more negative, and the adsorption capacities of MB, MV and Pb(II) substantially increased due to electrostatic attractions between the oppositely charged ions. When the solution pH reached to 6.0, the removal efficiency of MB and MV was 93.7% and 93.9%, respectively. Subsequent increase of solution pH did not obviously increase the adsorption capacities. In addition, the pH value of the original MB and MV solution was both measured to be approximately 6.0. Based on the removal efficiency and simple operation, subsequent experiments were carried out with the original MB and MV solution. However, when the pH values were higher than 6.0, hydrolysis of Pb(II) occurs, resulting in the formation of Pb(OH)_2_^35^. It would be difficult to distinguish between the adsorption and precipitation of Pb(II) removed from solutions. Therefore, a pH of 6.0 was chosen for subsequent adsorption experiments for Pb(II) to avoid precipitation of Pb(OH)_2_. As a result, the sorbent MWHPO-GO can both effectively remove the pollutants in acidic and basic solution. Therefore, we concluded that the prepared sorbent MWHPO-GO could be suitable for drinking water and waste-water.

### Effect of contact time on the adsorption behavior

The contact time between the adsorbent and adsorbate play an important role for evaluating the adsorption properties of adsorbents. Figure 3C,F show the influence of the contact time on the removal efficiency and adsorption capacities of MB, MV and Pb(II), respectively. In general, absorption is a time-consuming process. An increase in the contact time is advantageous for sufficient interactions between the pollutants and the adsorption sites of MWHPO-GO. For MB and MV adsorption, the removal efficiency increased sharply within 40 min and reached equilibrium in 150 min. For Pb(II) adsorption, the removal efficiency increased sharply within 30 min and reached equilibrium in 120 min. In the initial stage of the adsorption process, the adsorption sites on MWHPO-GO for the pollutants were sufficient. As the adsorption proceeds, the free binding sites become rarer, resulting in decrease in adsorption rate, and the adsorption capacity was eventually saturated. Therefore, 150 min and 120 min were selected as the optimum contact time for MB/MV and Pb(II) removal, respectively.

### Adsorption kinetics

Four adsorption equations were applied to describe the kinetics of the contaminant adsorbing onto MWHPO-GO^36^,^37^,^38^,^39^. Each model is expressed as follows:

Pseudo-first order model:


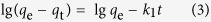


Pseudo-second order model:


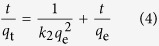


Intraparticle diffusion model:


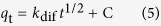


Bangham model:


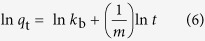


where *q*_e_ and *q*_t_ (mg g^−1^) are the amounts of pollutants adsorbed onto the adsorbent at equilibrium and at time *t* (min), respectively. *k*_1_ and *k*_2_ (mg min g^−1^) are the pseudo-first order and pseudo-second order rate constant, respectively. *k*_dif_ (mg g^−1^ min^−1/2^) is the intraparticle diffusion rate constant. *m* and *k*_b_ are the related constants of the Bangham model.

**Figure 4:**
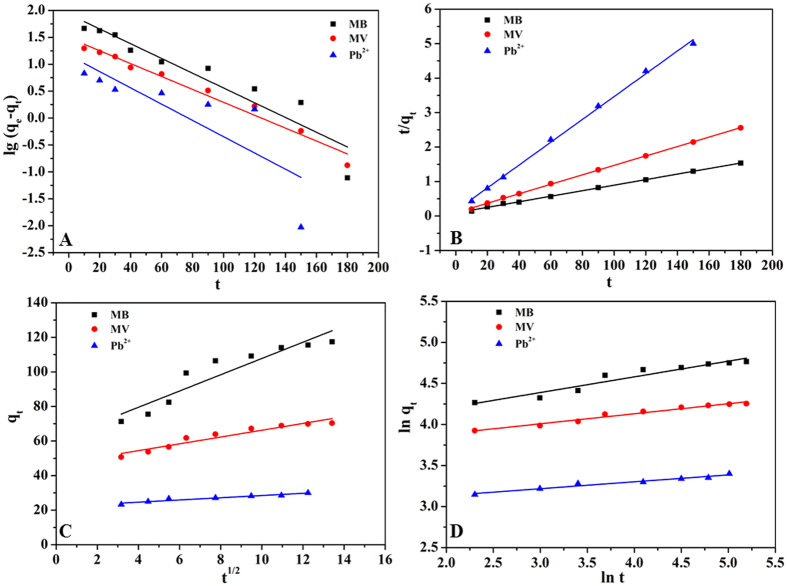
Pseudo-first-order kinetics (**A**), Pseudo-second-order kinetics (**B**), Intraparticle diffusion kinetics (**C**) and Bangham kinetics (**D**) for adsorption of MB (*m* = 6 mg, *C*_0_ = 30 mg L^−1^, *V* = 25 mL, pH = 6, temperature was 298 K), MV (*m* = 10 mg, *C*_0_ = 30 mg L^−1^, *V* = 25 mL, pH = 6, temperature was 298 K) and Pb(II) (*m* = 10 mg, *C*_0_ = 30 mg L^−1^, *V* = 10 mL, pH = 6, temperature was 298 K).

The linear fitting results of the kinetic data are shown in Fig. 4, and the relevant calculated results are listed in Supplementary Table S2 online. All of the experimental data fitted very well with the pseudo-second order kinetic model compared to the three other models (MB: R^2^ = 0.9985, MV: R^2^ = 0.9995, Pb(II): R^2^ = 0.9979), which implied that the adsorption rate was primarily controlled by chemisorption rather than mass transport^40^. In addition, the calculated *q*_e_ from the pseudo-second order kinetic model (124.4 mg g^−1^ for MB, 72.9 mg g^−1^ for MV and 30.2 mg g^−1^ for Pb(II)) are consistent with the experimental data (120.5 mg g^−1^ for MB, 70.5 mg g^−1^ for MV and 30.0 mg g^−1^ for Pb(II)).

### Adsorption isotherms

In order to provide insight into the adsorption behavior of an adsorbent, four isotherm equations were selected to model the adsorption isotherm data including the Henry^41^, Langmuir^42^, Freundlich^43^ and Dubinin-Radushkevich (D-R) equations^44^, which can be expressed as follows:

Henry equation:


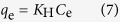


Langmuir equation:


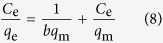


Freundlich equation:


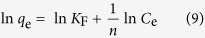


Dubinin-Radushkevich equation:


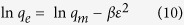


where *q*_m_ (mg g^−1^) is the maximum adsorption capacity. *K*_H_ and *K*_F_ are the constants related to the adsorption capacity and intensity, respectively. *b* (L mg^−1^) is the Langmuir constant related to the affinity of the binding site. A smaller 1/*n* value indicates a more heterogeneous surface. However, a value closer to or equal to one indicates the adsorbent has relatively more homogeneous binding sites. 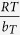 is related to the adsorption heat. *A*_*T*_ is the equilibrium constant corresponding to the maximum binding energy. *β* (mol^2^ kJ^−2^) is the average free energy generated per gram adsorbent. *ε* is Polanyi potential-energy.

**Figure 5:**
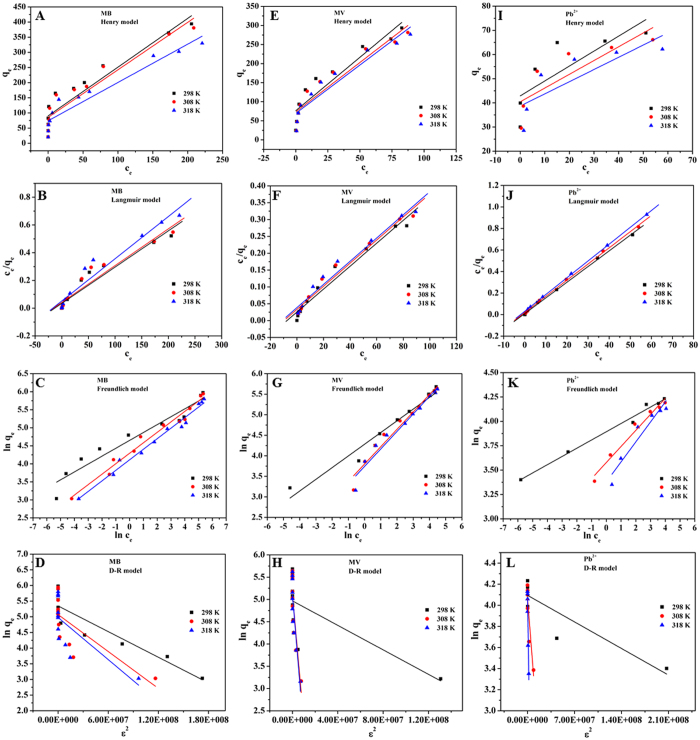
Henry (**A**), Langmuir (**B**), Freundlich (**C**) and D-R (**D**) adsorption isotherm fit of MB (*m* = 6 mg, *C*_0_ = 5–300 mg L^−1^, *V* = 25 mL, pH = 6, contact time was 150 min, temperature was 298 K, 308 K and 318 K, respectively); Henry (**E**), Langmuir (**F**), Freundlich (**G**) and D-R (**H**) adsorption isotherm fit of MV (*m* = 10 mg, *C*_0_ = 10–200 mg L^−1^, *V* = 25 mL, pH = 6, contact time was 150 min, temperature was 298 K, 308 K and 318 K, respectively) and Henry (**I**), Langmuir (**J**), Freundlich (**K**) and D-R (**L**) adsorption isotherm fit of Pb(II) (*m* = 10 mg, *C*_0_ = 30–120 mg L^−1^, *V* = 10 mL, pH = 6, contact time was 120 min, temperature was 298 K, 308 K and 318 K, respectively).

The fitted results of all isotherm models are presented in Fig. 5, and the calculated parameters are listed in Supplementary Table S3 online. On the basis of the correlation coefficients (R^2^), it can be seen that the Langmuir model is more suitable than the three other models for describing the adsorption of MB (R^2^ = 0.9670, R^2^ = 0.9648, R^2^ = 0.9742), MV (R^2^ = 0.9842, R^2^ = 0.9877, R^2^ = 0.9853) and Pb(II) (R^2^ = 0.9992, R^2^ = 0.9994, R^2^ = 0.9998) at 298 K, 308 K and 318 K, respectively. These results suggested that the surface of MWHPO-GO was covered by monolayer pollutant and there is no significant interaction among adsorbed species^45^. The maximum adsorption amount calculated from the Langmuir model was 381.7 mg g^−1^ for MB, 293.3 mg g^−1^ for MV and 68.7 mg g^−1^ for Pb(II) at 298 K. The 1/*n* value is an indicator of the favorite state of the absorption process. The smaller 1/*n* value (<0.5) indicated that MB, MV or Pb(II) was easily adsorbed onto the heterogeneous surface of the MWHPO-GO composite.

### Thermodynamic parameters

The thermodynamic studies provide in-depth information on inherent energetic changes during the adsorption process. In this work, the effects of the temperature on the adsorption were investigated and the thermodynamic behavior was evaluated using the following equations:


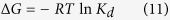



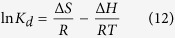


where *R* (8.314 J mol^−1^ K^−1^) is the gas constant, T (K) is the absolute temperature and *K*_d_ is the thermodynamic equilibrium constant. Δ*S* is the entropy change, Δ*H* is the enthalpy change, and Δ*G* is the Gibbs free energy change in a given process (kJ mol^−1^). The Δ*H*, Δ*S* and Δ*G* results are shown in Supplementary Table S4 online. The negative values of Δ*H* demonstrated the exothermic nature of the adsorption processes. The negative values of Δ*S* indicated that MB, MV or Pb(II) molecules were orderly adsorbed onto the MWHPO-GO. The negative values of Δ*G* suggested the spontaneous nature of the adsorption processes at the three temperatures.

### Adsorption mechanism

**Figure 6:**
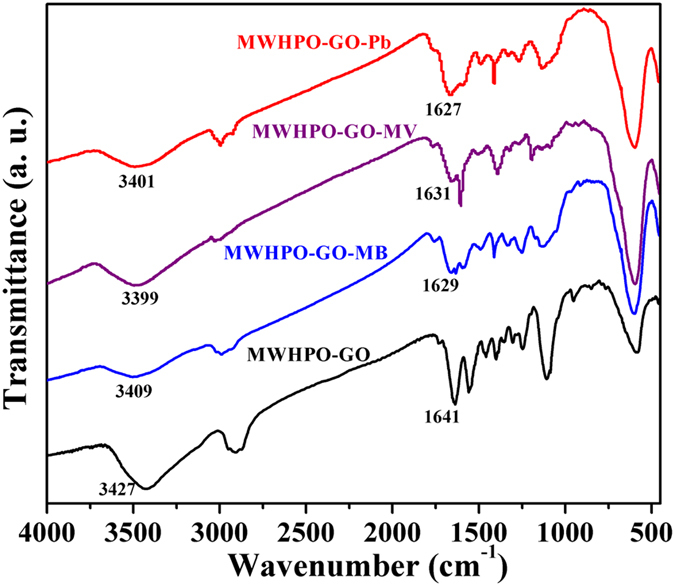
FTIR spectra of MWHPO-GO before and after loading MB (MWHPO-GO-MB), MV (MWHPO-GO-MV) and Pb(II) (MWHPO-GO-Pb).

**Figure 7:**
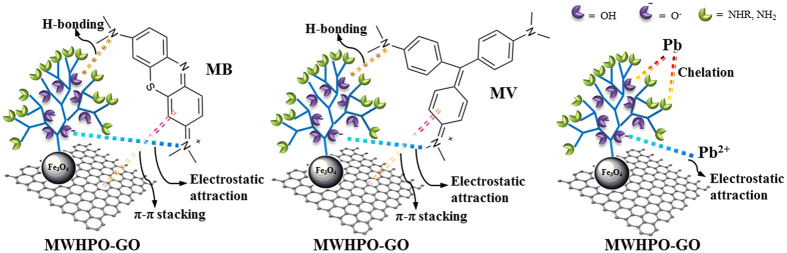
Proposed adsorption mechanism of MB, MV and Pb(II) adsorption onto MWHPO-GO.

In order to illustrate the adsorption mechanism, FTIR spectra of MWHPO-GO nanocomposites with adsorbed MB, MV and Pb(II) (referred to as MWHPO-GO-MB, MWHPO-GO-MV and MWHPO-GO- Pb(II), respectively) were recorded to study the possible interaction sites between adsorbent and adsorbate molecule (Fig. 6). In the spectra characteristic peak shifts were observed. For MB/MV removal, the peak at 3427 cm^−1^ corresponding to the -OH and -NH_2_ stretching vibration shifted to 3409 cm^−1^ (for MB) and 3399 cm^−1^ (for MV), indicating the electrostatic attractions as well as H-bonding interaction between the active sites of cationic MB and MV dyes and the hydroxyls groups and amine groups of MWHPO-GO nanocomposites^9^,^46^. Besides, the adsorption bands 1641 cm^−1^ corresponding to the aromatic rings vibration and N-H bending vibration shifted to 1629 cm^−1^ (for MB) and 1631 cm^−1^ (for MV), suggesting the existence of H-bonding interaction and π-π stacking interactions may occur between the aromatic rings of MB/MV molecules and MWHPO-GO^47^,^48^,^49^. For Pb(II) removal, the characteristics peaks shifted for the stretching vibration of the -OH (or -NH) groups from 3427 cm^−1^ to 3401 cm^−1^, the bending vibration of the -NH_2_ groups from 1641 cm^−1^ to 1627 cm^−1^, indicating the electrostatic attractions as well as chelation interaction between the amino and hydroxyl groups of MWHPO-GO and lead^50^,^51^. A possible mechanism has been proposed in Fig. 7.

### Performance evaluation

The effect of common metal ions, such as K^+^, Na^+^, Ca^2+^ and Mg^2+^, on the dyes and heavy metal adsorption was investigated using KNO_3_, NaNO_3_, Ca(NO_3_)_2_ and Mg(NO_3_)_2_ as the ionic medium. In the present study, K^+^, Na^+^, Ca^2+^ and Mg^2+^ solutions with different concentration were utilized at otherwise constant parameters (adsorbent dosage 6 mg, volume 25 mL, pH 6, contact time 150 min, temperature 298 K, dye concentration 30 mg L^−1^ for MB, adsorbent dosage 10 mg, volume 25 mL, pH 6, contact time 150 min, temperature 298 K, dye concentration 30 mg L^−1^ for MV and adsorbent dosage 10 mg, volume 25 mL, pH 6, contact time 120 min, temperature 298 K, heavy metal concentration 30 mg L^−1^ for Pb(II)). Their presence may compete with MB, MV or Pb(II) for binding at the adsorption sites on the surface of MWHPO-GO^52^. Based on the results shown in Supplementary Fig. S3 online, the adsorption capacities of the three pollutants decreased slightly along with the increased coexisting ions, which implied that the high-concentration coexisting ions had weak interferences to the MB, MV and Pb(II) adsorption. However, the decrease is still acceptable compared to the high removal efficiency of the contaminants.

**Figure 8:**
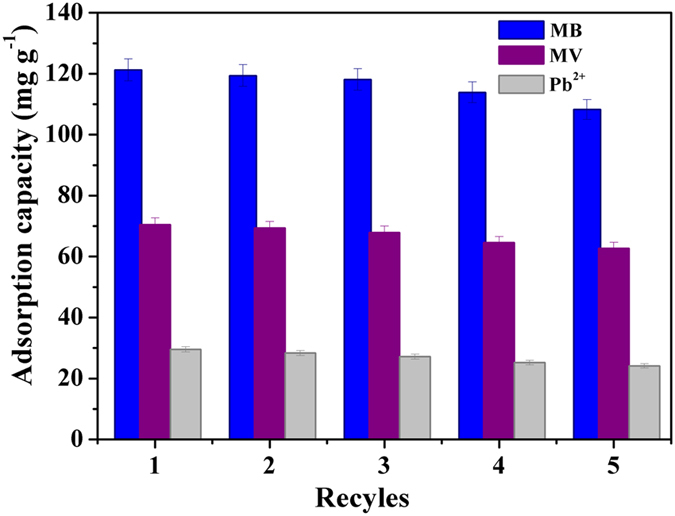
Adsorption-desorption recycles of MB (*m* = 6 mg, *C*_0_ = 30 mg L^−1^, *V* = 25 mL, pH = 6, contact time was 150 min, temperature was 298 K), MV (*m* = 10 mg, *C*_0_ = 30 mg L^−1^, *V* = 25 mL, pH = 6, contact time was 150 min, temperature was 298 K) and Pb(II) (*m* = 10 mg, *C*_0_ = 30 mg L^−1^, *V* = 10 mL, pH = 6, contact time was 120 min, temperature was 298 K). Error bar = SD (n = 2).

The recycling and regeneration abilities of the adsorbent are crucial for evaluating their performance for practical applications. In this study, low-cost reagents (HCl solutions (2 M) and ethanol) were found to be effective desorption agents to recover MB, MV and Pb(II), respectively, from the MWHPO-GO adsorbent. As shown in Fig. 8, after 5 times of adsorption–desorption recycling experiments, MWHPO-GO still exhibited acceptable adsorption capacities for these three pollutants with low decline. In addition, it should not be forgotten that the MWHPO-GO adsorbent can be facilely recycled by a magnet. Therefore, MWHPO-GO promised a great potential for easy recycling and reuse for wastewater treatment.

In addition, the maximum adsorption capacities of MWHPO-GO nanocomposites for MB, MV and Pb(II) were compared with those of other reported adsorbents to illustrate the excellent adsorption performance of MWHPO-GO. As can be seen form Supplementary Table S5 online, the maximum adsorption capacities of MWHPO-GO for MB, MV and Pb(II) were higher than those of other adsorbents. This can be explained from two aspects: First, Fe_3_O_4_ nanoparticles and GO nanosheets have a high specific surface area, which increases the contact area between the adsorbent and adsorbate. Second, the existence of water-soluble hyperbranched polyol endowed MWHPO-GO with good water dispersity and abundant hydroxyl and amine groups, which can strengthen the interaction between the adsorbent and the adsorbate. Therefore, MWHPO-GO can effectively improve the adsorption capacity of MB, MV and Pb(II). Considering the convenient separation process and good adsorption performance, MWHPO-GO is a good absorbent for polluted water treatment.

## Conclusions

In this study, a novel nanocomposite magnetic adsorbent based on surface modification of graphene oxide with water-soluble hyperbranched polyol grafted magnetic particles was successfully prepared, which showed well removal properties of MB, MV and Pb(II) from aqueous solutions. MWHPO-GO adsorbent can be facilely recycled by a magnet. The pseudo-second order equation and Langmuir adsorption isotherm model fitted well with the kinetics data and isotherm results obtained from the adsorption processes of these three pollutants, respectively. In addition, the thermodynamic studies illustrated that the adsorption process was exothermic and spontaneous in nature. It was important that MWHPO-GO can be effectively regenerated by low-cost reagents and retained considerable adsorption capacity after several adsorption-desorption cycles. MWHPO-GO exhibited a great potential for water purification based on the high efficiency and feasibility.

## Additional Information

**How to cite this article**: Hu, L. *et al*. Fabrication of magnetic water-soluble hyperbranched polyol functionalized graphene oxide for high-efficiency water remediation. *Sci. Rep.* **6**, 28924; doi: 10.1038/srep28924 (2016).

## Supplementary Material

Supplementary Information
