# Supplementary material for: Fabrication of magnetic water-soluble hyperbranched polyol functionalized graphene oxide for high-efficiency water remediation
Source: Sci Rep. 2016 Jun 29;6:28924. doi: 10.1038/srep28924 (PMC4926210; doi:10.1038/srep28924)
Supplement: Supplementary Information [file srep28924-s1.pdf]

Supplementary information

Fabrication of magnetic water-soluble hyperbranched polyol functionalized graphene oxide for high-efficiency water remediation

Lihua Hu [a], Yan Li [a], Xuefei Zhang [a], Yaoguang Wang [a], Limei Cui [b], Qin Wei [a], Hongmin Ma [a], Liangguo Yan [b], Bin Du [b]*

[a] Key Laboratory of Chemical Sensing & Analysis in Universities of Shandong, School of Chemistry and Chemical Engineering, University of Jinan, Jinan 250022, PR China

[b] School of Resources and Environment, University of Jinan, Jinan 250022, PR China

*Corresponding author. Tel. + 86-531-82767370; fax: + 86-531-82767370.

E-mail address: dubin61@gmail.com (Bin Du).

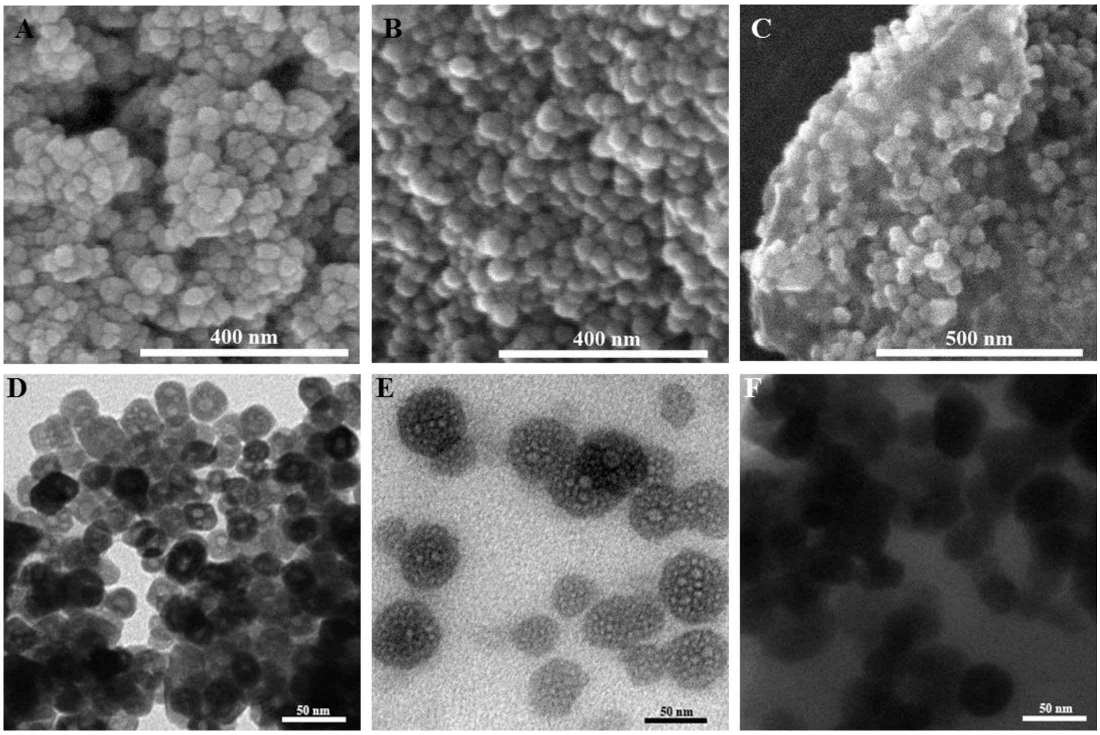

Figure S1. SEM images of Fe_3O_4 (A), MWHPO (B) and MWHPO-GO (C); TEM images of Fe_3O_4 (D), MWHPO (E) and MWHPO-GO (F).

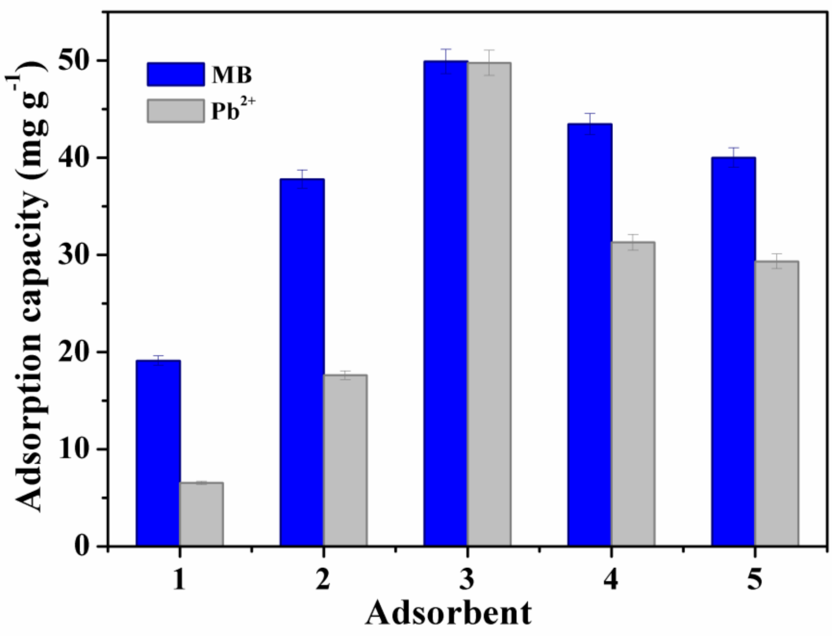

Figure S2. Adsorption capacities of MB and Pb(II) by using MWHPO (number 1), MWHPO-GO-0.5 (number 2), MWHPO-GO (number 3), MWHPO-GO-3 (number 4) and MWHPO-GO-5 (number 5) adsorbent, respectively. (C_0 = 20 mg L^{-1}, V = 25 mL, dosage = 10 mg, contact time was 3 h, temperature was 298 K). Error bar = SD (n = 2).

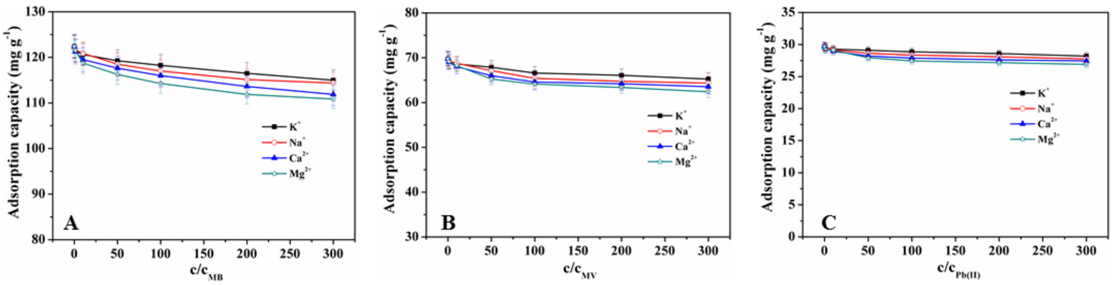

Figure S3. Effect of coexisting ions on adsorption of MB (m = 6 mg, C_{MB} = 30 mg L^{1}, V = 25 mL, pH = 6, contact time was 150 min, temperature was 298 K), MV (m = 10 mg, C_{MV} = 30 mg L^{-1}, V = 25 mL, pH = 6, contact time was 150 min, temperature was 298 K) and Pb(II) (m = 10 mg, $C_{Pb(II)}$ = 30 mg L^{-1}, V = 10 mL, pH = 6, contact time was 120 min, temperature was 298 K). The concentration ratio of coexisting ions (K^+, Na^+, Ca^{2+} or Mg^{2+}) to MB (c/c_{MB}), MV (c/c_{MV}) or Pb(II) ($c/c_{Pb(II)}$) was 0, 1, 10, 50, 100, 200 and 300, respectively. Error bar = SD (n = 2).

Table S1. Weight percentage of elements obtained from EDX analysis.

Sample name	Weight percentage of elements (%)				
	C	O	Fe	S	Pb
MWHPO-GO	23.79	28.91	47.30	-	-
MWHPO-MB	35.80	19.80	43.16	1.24	-
MWHPO-Pb(II)	28.87	26.81	37.26	-	7.06

Table S2. Constants and correlation coefficients for the kinetic models of MB, MV and Pb(II) onto MWHPO-GO.

Kinetic model	Parameter	MB	R^2	MV	R^2	Pb(II)	R^2
Pseudo-first-order	k_1 (mg min g^{-1})	0.0137	0.8793	0.0119	0.9736	0.015	0.6134
	q_e (mg g^{-1})	85.16		31.01		14.67	
Pseudo-second-order	k_2 (mg min g^{-1})	0.0007	0.9985	0.0019	0.9995	0.0071	0.9979
	q_e (mg g^{-1})	124.38		72.94		30.22	
Intraparticle diffusion	k_{dif} (mg g^{-1} $min^{-1/2}$)	4.7066	0.8654	1.9678	0.9164	0.6407	0.9183
Bangham	m	5.2359	0.9216	8.1800	0.9706	15.5836	0.9545
	k_b (mg g^{-1})	245.4194		38.1498		19.4011	

Table S3. Adsorption isotherm parameters for MB, MV and Pb(II) adsorption onto MWHPO-GO at 298 K, 308 K, 318 K.

Temperature	Adsorption isotherm	Parameter	MB	R^2	MV	R^2	Pb(II)	R^2
298 K	Henry	K_H	1.6093	0.8846	2.7997	0.8850	0.6180	0.5764
	Langmuir	b (L mg^{-1})	0.0757	0.9670	0.1342	0.9842	1.455	0.9992
		q_m (mg g^{-1})	381.68		293.26		68.73	
	Freundlich	K_F	104.58	0.9329	72.40	0.9542	49.08	0.9859
		$1/n$	0.2187		0.2849		0.0845	
	D–R	β (mol^2 kj^{-2})	1.4×10^{-8}	0.8195	1.4×10^{-8}	0.4296	3.6×10^{-9}	0.7524
		q_m (mg g^{-1})	209.72		143.65		60.02	
308 K	Henry	K_H	1.5681	0.8888	2.5918	0.8864	0.5698	0.6404
	Langmuir	b (L mg^{-1})	0.0634	0.9648	0.1028	0.9877	0.7524	0.9994
		q_m (mg g^{-1})	377.36		291.55		66.62	
	Freundlich	K_F	73.18	0.9724	44.67	0.9561	35.80	0.9658
		$1/n$	0.2899		0.4228		0.1647	
	D–R	β (mol^2 kj^{-2})	2.0×10^{-8}	0.5011	2.8×10^{-7}	0.7328	8.1×10^{-8}	0.7797
		q_m (mg g^{-1})	159.26		165.02		58.49	
318 K	Henry	K_H	1.2652	0.9143	2.5313	0.9006	0.5049	0.6032
	Langmuir	b (L mg^{-1})	0.0589	0.9742	0.0898	0.9853	0.4927	0.9998
		q_m (mg g^{-1})	327.87		289.86		64.19	
	Freundlich	K_F	61.06	0.9797	42.19	0.9577	29.36	0.9071
		$1/n$	0.2924		0.4258		0.2048	
	D–R	β (mol^2 kj^{-2})	2.2×10^{-8}	0.7059	3.1×10^{-7}	0.7472	4.0×10^{-7}	0.8938
		q_m (mg g^{-1})	145.05		163.65		57.26	

Table S4. Thermodynamic parameters for MB, MV and Pb(II) adsorption onto MWHPO-GO.

Pollutants	Temperature	lnK_d	ΔG (kJ mol^{-1})	ΔH (kJ mol^{-1})	ΔS (J mol^{-1} K^{-1})
	298 K	2.072038	-5.134		
MB	308 K	1.749388	-4.480	-16.367	-37.989
	318 K	1.658933	-4.386		
	298 K	1.750844	-4.338		
MV	308 K	1.441750	-3.692	-13.702	-31.775
	318 K	1.405852	-3.717		
	298 K	2.733496	-6.772		
Pb(II)	308 K	2.019710	-5.172	-40.070	-112.250
	318 K	1.720435	-4.549		

Table S5. Asorption capacities of various adsorbents for MB, MV and Pb(II).

Type of adsorbent	Target element	q_m (mg g^{-1})	Ref.
MWHPO-GO	MB	381.7	This paper
	MV	293.3	
	Pb(II)	68.7	
MGO	MB	64.2	1
PANF-g-HPEI	MB	161	2
MCGO	MB	95.1	3
Graphene	MB	153.9	4
Baker's yeast modified by nano-Fe_3O_4	MV	60.8	5
CSM	MV	99.3	6
MCDBs	MV	61.1	7
Pb-IIPs	Pb(II)	9	8
Amino-functionalized Fe_3O_4	Pb(II)	40	9
Graphene nanosheets	Pb(II)	35.7	10
PDA-GO	Pb(II)	53.6	11

1. Deng, J.H. et al. Simultaneous removal of Cd(II) and ionic dyes from aqueous solution using magnetic graphene oxide nanocomposite as an adsorbent. *Chem. Eng. J.* **226**, 189-200 (2013).

2. Cai, X.Q. et al. Novel Pb^{2+} ion imprinted polymers based on ionic interaction via synergy of dual functional monomers for selective solid-phase extraction of Pb^{2+} in water samples. *Acs Appl. Mater. Interfaces* **6**, 305-313 (2014).

3. Tan, Y.Q., Chen, M. & Hao, Y.M. High efficient removal of Pb (II) by amino-functionalized Fe_3O_4 magnetic nano-particles. *Chem. Eng. J.* **191**, 104-111 (2012).

4. Fan, Y., Liu, H.J., Zhang, Y. & Chen, Y. Adsorption of anionic mo or cationic mb from mo/mb mixture using polyacrylonitrile fiber hydrothermally treated with hyperbranched polyethylenimine. *J. Hazard. Mater.* **283**, 321-328 (2015).

5. Fan, L.L. et al. Fabrication of novel magnetic chitosan grafted with graphene oxide to enhance adsorption properties for methyl blue. *J. Hazard. Mater.* **215-216**, 272-279 (2012).

6. Liu, T.H. et al. Adsorption of methylene blue from aqueous solution by graphene. *Colloids Surface B* **90**, 197-203 (2012).

7. Tian, Y. et al. Preparation and characterization of baker's yeast modified by nano-Fe_3O_4: application of biosorption of methyl violet in aqueous solution. *Chem. Eng. J.* **165**, 474-481 (2010).

8. Lin, Q.T., Pan, J.X., Lin, Q.L. & Liu, Q.J. Microwave synthesis and adsorption performance of a novel crosslinked starch microsphere. *J. Hazard. Mater.* **263**, 517-524 (2013).

9. Li, Y., Xiao, H.N., Chen, M.D., Song, Z.P. & Zhao, Y. Absorbents based on maleic anhydride-modified cellulose fibers/diatomite for dye removal. *J. Mater. Sci.* **49**, 6696-6704 (2014).

10. Huang, Z.H. et al. Adsorption of lead(II) ions from aqueous solution on low-temperature exfoliated grapheme nanosheets. *Langmuir* **27**, 7558-7562 (2011).

11. Dong, Z.H. et al. Bio-inspired surface-functionalization of grapheme oxide for the adsorption of organic dyes and heavy metal ions with a superhigh capacity. *J. Mater. Chem. A* **2**, 5034-5040 (2014).
